# Multivariable analysis of factors associated with USMLE scores across U.S. medical schools

**DOI:** 10.1186/s12909-019-1605-z

**Published:** 2019-05-20

**Authors:** Arash Ghaffari-Rafi, Rachel Elizabeth Lee, Rui Fang, J. Douglas Miles

**Affiliations:** 1 University of Hawai‘i at Mānoa John A. Burns School of Medicine, Honolulu, Hawai‘i USA; 2 Queen Square Institute of Neurology, University College London, London, UK

**Keywords:** United States medical licensing examination, USMLE, Evaluation, Student learning, Curriculum, Assessment

## Abstract

**Background:**

Gauging medical education quality has always remained challenging. Many studies have examined predictors of standardized exam performance; however, data sets do not distinguish by institution or curriculum. Our objective is to present a summary of variables associated with the United States Medical Licensing Examination (USMLE) scores, and thus identify institutions (and therefore curriculums) which deviate from trend lines by producing higher USMLE scores despite having lower entrance grade point averages and medical college admissions test (MCAT) scores.

**Methods:**

Data was obtained from U.S. News and World Report’s 2014 evaluation of allopathic U.S. medical schools. A univariate analysis was performed first for each variable using two sample t-test or Wilcoxon rank sum test for categorical variables, and Pearson or Spearman correlation coefficients for continuous variables. A multivariable linear regression model was developed to identify the factors contributing to USMLE scores. All statistical analyses were two-sided and performed using SAS software version 9.4 (SAS Institute Inc., Cary, NC).

**Results:**

Univariate analysis reveals a significant association between USMLE Step 1 and 2 scores with medical college admissions test scores, grade point averages, school type (private vs. public), full-time faculty-to-student ratio, National Institute of Health funds, residency director assessment score, peer assessment score, and class size. Of these nine variables, MCAT scores and Step 1 scores display the strongest correlation (corr = 0.72, *P* < .0001). Multivariable analysis also supports a significant association between MCAT scores and Step scores, meanwhile National Institute of Health funding size demonstrates a negative correlation with USMLE Step 2 scores. Although MCAT scores and National Institute of Health funds are significantly associated with USMLE performance, six outlier institutions were identified, producing higher USMLE scores than trend line predictions.

**Conclusions:**

Outlier institutions produce USMLE scores that do not follow expected trend lines. Their performance might be explainable by differences in curriculum. Having identified these institutions, their curriculums can be further studied to determine what factors enhance student learning.

## Background

Gauging medical education quality has always remained challenging due to the myriad of factors that can be assessed, including those which are difficulty to quantify—such as adherence to the medical school’s mission statement. Despite such challenges, prior medical school assessments have emphasized school admissions rate, entering class Medical College Admissions Test (MCAT) and grade point averages (GPA), full-time faculty-to-student ratio, and National Institute of Health (NIH) funding [1–3]. Meanwhile, two forms of student evaluation that occur during the time of medical studies include assessments in clinical clerkships and United States Medical Licensing Examination (USMLE) exams; due to variability in scoring systems for clinical clerkships, the most consistent measurement of school product is the USMLE Step exams [4]. Step 1 assesses basic science knowledge, whereas Step 2 focuses on clinical understanding [4]. These exams are the primary academic criteria for residency selection, for to an extent they provide a gauge of student learning [5, 6].

Many studies have examined predictors of standardized exam performance; however, data sets do not distinguish by institution or curriculum (i.e., problem based learning, lectures, team based learning, etc.). Moderate correlations have been identified between USMLE Step 1, MCAT, and undergraduate GPA [7–11]. Performance on Step 2 Clinical Knowledge (CK) exam has also been associated with performance on USMLE Step 1 and the MCAT [12–15]. However, numerous predictors of USMLE performance, including subjective predictors (i.e. peer assessment score), have not been compared against objective predictors (i.e. standardized exam scores), and thus, their reliability is unknown. This study examines multiple variables to determine which factors play a greater role in determining medical student success, as well as identifies institutions that significantly deviate from expected trend lines, and thus identify those curricula that may potentially excel in efficiently educating students.

## Methods

### Design and setting

Data was collected from a publicly accessible database, U.S. News and World Report’s (USN&WR), and does not contain specific student identifiers. Institutional review board exemption for waivers of informed consent was attained from the University of Hawai‘i at Mānoa, Office of Research Compliance. Permission to utilize data from USN&WR in a non-commercial manner was attained from the Permissions Office and the Director of Specialty Marketing at USN&WR. Only publicly available data was utilized in our analysis.

USN&WR (https://www.usnews.com/best-graduate-schools/top-medical-schools/research-rankings) surveyed 130 medical schools fully accredited by the Liaison Committee on Medical Education. Of those schools, 100 provided data. 2014 data was compiled to compare average USMLE Step 1 and Step 2 scores against nine variables: median undergraduate GPA, median MCAT, school type (private vs public), full-time faculty-to-student ratio, NIH funds granted to the medical school and affiliated hospitals, NIH research grant funds per faculty member, peer assessment score, residency directors assessment, and total medical school enrollment.

Median MCAT total scores and undergraduate GPAs were obtained from students taking USMLE in 2014. Faculty resources were measured as the ratio of full-time science and full-time clinical faculty to full-time M.D. students. Research activity was based on the total dollar amount of grants awarded by the NIH to the medical school and its affiliated hospitals, and of NIH grant funding per full-time faculty member.

The peer assessment score was based on subjective ratings collected from medical school deans, deans of academic affairs, department heads of internal medicine, and directors of admissions from other medical schools. These respondents rated programs on a scale from 1 (marginal) to 5 (outstanding). For fair evaluation, individuals with limited knowledge about a medical school were requested to select the neutral response “don’t know,” from the scale of response options. A school’s average score was the average rating of all the respondents who rated it. Residency program directors were also asked to rate programs using the same 5-point scale. Each medical school reported total medical school enrollment in year 2014 to USN&WR.

### Statistical analysis

The data was summarized by descriptive statistics: mean with standard deviation (SD) or median with minimum and maximum for continuous variables (based on distribution) such as Step scores, and frequency and percentage for categorical variables such as school type (public or private). To access the association with Step scores, a univariate analysis was performed first for each variable using two sample t-test or Wilcoxon rank sum test for categorical variables, and Pearson or Spearman correlation coefficients for continuous variables. A multivariable linear regression model was developed to identify the factors contributing to USMLE scores. Significant variables in the univariate analysis were considered to be included into the model. All statistical analyses were two-sided and performed using SAS software version 9.4 (SAS Institute Inc., Cary, NC). An alpha level of 0.05 was used to determine statistical significance.

## Results

100 U.S. medical schools reported both USMLE Step 1 and 2 scores, and thus are the focus of this analysis. Average Step 1 and 2 scores are 230.5 (SD = 6.0) and 240.0 (SD = 4.9), respectively. Factors that associate with USMLE scores are summarized in Table 1. Fifty-nine (59.0%) of schools are public. On average, the median GPA and MCAT scores are 3.7 (SD = 0.09) and 32.1 (SD = 2.6), respectively. The median full-time faculty-student ratio is 1.8 (ranged from 0.2 to 14.9). The median NIH funds granted to the medical school and affiliated hospitals are 88.9 million (ranged from 1.8 to 1412.9 million). The median NIH research funds per faculty member are 87.47 thousand (ranged from 4.57 to 381.84 thousand). On average, the residency directors’ assessment score is 3.4 (SD = 0.6) and the peer assessment score is 3.1 (SD = 0.7). The median of total medical school enrollment in the year 2014 is 631.5 (ranged from 216 to 1377).

**Table 1:** Summary of USMLE scores and potential factors

Variable	
Avg. STEP1 score, mean (SD)	230.5 (6.0)
Avg. STEP2 score, mean (SD)	240.0 (4.9)
School Type, n (%)	
Private	41 (41.0%)
Public	59 (59.0%)
Median GPA, mean (SD)	3.7 (0.09)
Median MCAT total score, mean (SD)	32.1 (2.6)
Full-Time Faculty-Student Ratio, median (min, max)	1.8 (0.2, 14.9)
NIH Funds Granted to Medical School and Affiliated Hospitals in millions, median (min, max)	88.9 (1.8, 1412.9)
NIH Research Grant Funds Per Faculty Member in thousands, median (min, max)	87.47 (4.57, 381.84)
Residency Directors Assessment Score out of 5, mean (SD)	3.4 (0.6)
Peer Assessment Score out of 5, mean (SD)	3.1 (0.7)
Total Medical School Enrollment, median (min, max)	631.5 (216, 1377)

The association between USMLE scores and potential factors are summarized in Table 2. There are statistically significant correlations between average Step 1 score and median GPA (corr = 0.55, *P* < .0001), median MCAT total score (corr = 0.72, *P* < .0001), full-time faculty-to-student ratio (corr = 0.47, *P* < .0001), NIH funds granted to medical schools and affiliated hospitals (corr = 0.58, *P* < .0001), NIH research grant funds per faculty member (corr = 0.54, *P* < .0001), residency directors assessment score (corr = 0.60, *P* < .0001), and peer assessment score (corr = 0.62, *P* < .0001). There is a significant difference between private and public schools in Step 1 scores (*P* < .0001). On average, private schools have around a five-point higher average Step 1 score, compared to public schools (233.2 vs. 228.6). Regarding average Step 2 scores, there are statistically significant correlations with Step 1 score (corr = 0.54, *P* < .0001), median GPA (corr = 0.49, *P* < .0001), median MCAT total score (corr = 0.60, *P* < .0001), full-time faculty-to-student ratio (corr = 0.35, *P* = 0.0004), NIH funds granted to medical schools and affiliated hospitals (corr = 0.46, *P* < .0001), NIH research grant funds per faculty member (corr = 0.35, *P* = 0.0005), residency directors assessment score (corr = 0.47, *P* < .0001), and peer assessment score (corr = 0.49, *P* < .0001). Compared to public schools, private schools have a slightly higher Step 2 score (241.3 vs. 239.2, *P* = 0.051).

**Table 2:** Results of Univariate analysis with USMLE scores

Variable	Avg. STEP1 score	*P*-value	Avg. STEP2 score	*P*-value
Avg. STEP1 score	NA	NA	0.54	<.0001
Median GPA	0.55	<.0001	0.49	<.0001
Median MCAT total score	0.72	<.0001	0.60	<.0001
School type, mean (SD)		<.0001		0.051
Private	233.2 (6.3)		241.3 (5.7)	
Public	228.6 (5.0)		239.2 (4.1)	
Full-Time Faculty-Student Ratio	0.47	<.0001	0.35	0.0004
NIH Funds Granted to Medical School and Affiliated Hospitals in millions	0.58	<.0001	0.46	<.0001
NIH Research Grant Funds Per Faculty Member in thousands	0.54	<.0001	0.35	0.0005
Residency Directors Assessment Score out of 5	0.60	<.0001	0.47	<.0001
Peer Assessment Score out of 5	0.62	<.0001	0.49	<.0001
Total Medical School Enrollment	−0.10	0.33	0.02	0.86

Variables with a significant bivariate relationship to Step 1 score were entered into a linear model to predict Step 1 score. These variables include: median GPA, median MCAT total score, school type, full-time faculty-to-student ratio, NIH funds granted to medical schools and affiliated hospitals, NIH research grant funds per faculty member, residency director assessment score and peer assessment score. Results are presented in Table 3. The results of the regression indicate that eight variables explained 58.4% of the variance (R^2^ = 0.584, *P* < .0001). Higher median MCAT significantly predicted higher Step 1 score (β = 1.28, *P* = 0.0002).

**Table 3:** Results of multivariable linear regression model for STEP 1 score

Variable	Parameter Estimate	Standard Error	*P*-value
Intercept	153.58	21.83	<.0001
Median GPA	10.01	6.84	0.15
Median MCAT total score	1.28	0.33	0.0002
School type, Private vs. Public	1.36	1.01	0.19
Full-Time Faculty-Student Ratio	−0.17	0.30	0.57
NIH Funds Granted to Medical School and Affiliated Hospitals in millions	0.0004	0.0042	0.93
NIH Research Grant Funds Per Faculty Member in thousands	0.002	0.01	0.85
Residency Directors Assessment Score out of 5	−3.82	2.77	0.18
Peer Assessment Score out of 5	3.61	2.78	0.20

Variables with a significant bivariate relationship to Step 2 score were entered into a linear model to predict Step 2 score. These variables include: average Step 1 score, median GPA, median MCAT total score, school type, full-time faculty-to-student ratio, NIH funds granted to medical schools and affiliated hospitals, NIH research grant funds per faculty member, residency director assessment score, and peer assessment score. Results are present in Table 4. The results of the regression indicate that nine variables explained 46.9% of the variance (R^2^ = 0.469, *P* < .0001). Change of the following variables significantly predicts higher Step 2 scores: higher median MCAT total score (β = 1.11, *P* = 0.012) and lower NIH research grant funds per faculty member (β = − 0.02, *P* = 0.039).

**Table 4:** Results of multivariable linear regression model for STEP 2 score

Variable	Parameter Estimate	Standard Error	*P*-value
Intercept	148.45	25.3	<.0001
Avg. STEP1 score	0.13	0.10	0.20
Median GPA	7.30	6.33	0.26
Median MCAT total score	1.10	0.33	0.0012
School type, Private vs. Public	−0.99	0.94	0.30
Full-Time Faculty-Student Ratio	−0.147	0.27	0.87
NIH Funds Granted to Medical School and Affiliated Hospitals in millions	0.002	0.0038	0.53
NIH Research Grant Funds Per Faculty Member in thousands	−0.02	0.009	0.039
Residency Directors Assessment Score out of 5	1.61	2.56	0.53
Peer Assessment Score out of 5	−1.50	2.56	0.56

### Additional analysis to identify outlier and influential points (fit diagnostic)

**Fig. 1:**
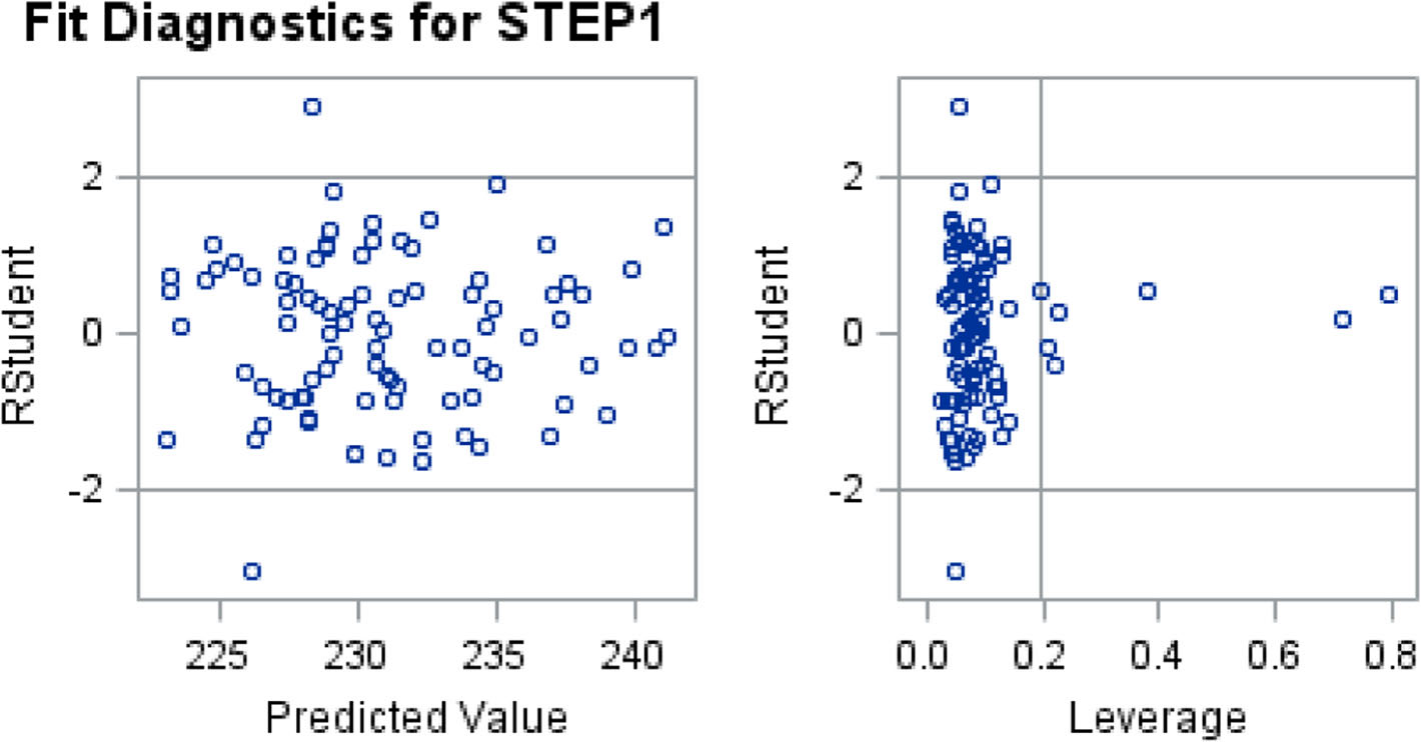
Fit Diagnostics for STEP1

**Fig. 2:**
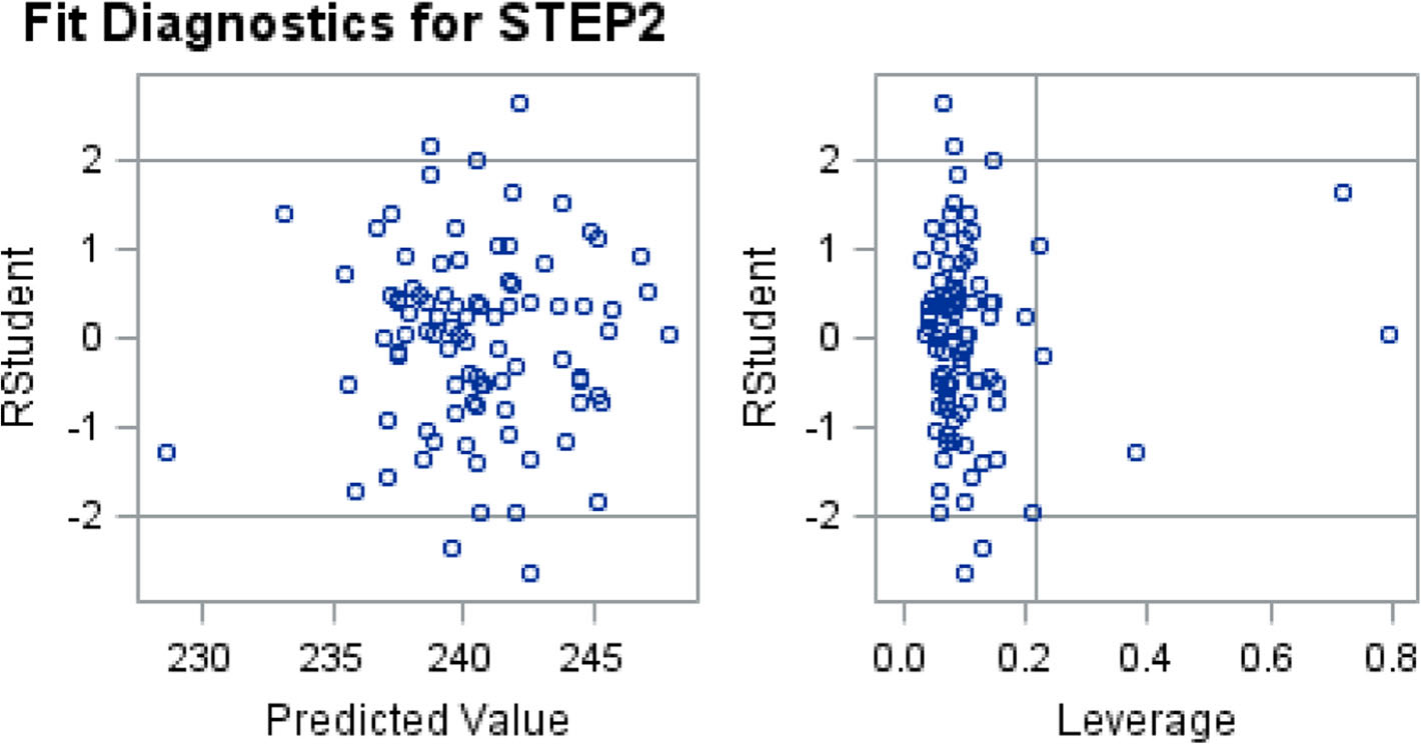
Fit Diagnostics for STEP2

The studentized residual (r) and leverage (lev) were assessed to identify the schools that are potential outliers or have potential influences on regression coefficients estimates. For multivariable linear model for Step 1 score, the potential outliers are University of Missouri-Columbia School of Medicine (*r* = 2.944) and University of Arkansas (*r* = − 3.089); the potential influence points are Harvard University with the largest leverage value of 0.792, followed by Mayo Medical School (lev = 0.715), Morehouse School of Medicine (lev = 0.378), University of Washington (lev = 0.229), New York University (lev = 0.222), and Stanford University (lev = 0.208). For the multivariable linear model for Step 2 score, the potential outliers are Emory University (*r* = 2.662), University of North Carolina (*r* = 2.164), University of Missouri-Columbia School of Medicine (*r* = 2.002), Uniformed Service University of the Health Sciences (*r* = − 2.345), and Duke University (*r* = − 2.646), and the potential influence points are Harvard University (lev = 0.793), Mayo Medical School (lev = 0.715), Morehouse School of Medicine (lev = 0.381), University of Washington (lev = 0.231) and New York University (lev = 0.223). In Figs. 1 and 2, the observations outside two horizontal lines are potential outliers and the observations beyond the vertical line are potential influences.

## Discussion

2014 dataset collected by USN&WR are comparable to publically available Association of American Medical Colleges (AAMC) data [16, 17]. The average AAMC Step 1 score of 229 (SD = 20) is comparable to USN&WR average of 230.5 (SD = 6.0), AAMC median GPA of 3.69 (SD = 0.25) is comparable to USN&WR GPA of 3.7 (SD = 0.09), and AAMC median MCAT score of 31.4 (SD = 3.9) is comparable to USN&WR MCAT of 32.1 (SD = 2.6). Variables unique to USN&WR data are residency director assessment score and peer assessment score.

Univariate analysis (Table 2) suggests that all measured variables except total medical student enrollment are significant predictors of Step 1 and Step 2 scores, with MCAT having the highest correlation. Such corresponds with other studies utilizing different data sets, which indicate that MCAT is a strong predictor of medical school success, and thus positively correlates with Step scores [8, 18]. On the other hand, school type is a marginally significant predictor of Step 2 scores as compared to Step 1 scores. One possible explanation for the difference between public and private medical schools, is that public institutions attain significant state funding. Therefore, states have an impetus to ensure that public medical schools are socially accountable by producing the much-needed primary care practitioners; hence accounting for public schools producing graduates who are more likely to choose primary care careers versus students trained in private medical schools [19]. With a greater likelihood of pursuing primary care, students in public institutions are less likely to pursue specialties which require more competitive Step scores, thus by extension yielding in public schools having slightly lower scores [20].

The only significant variable in the multivariable regression analysis model for Step 1 score is median MCAT score (Table 3), whereas NIH research grant funds per faculty member are an additional significant variable associated with Step 2 scores (Table 4). Surprisingly, the amount of grant funding schools received correlated inversely with Step 2 scores. There may be various explanations for this: perhaps the faculty at schools without abundant grant funding spend less time on research and more in patient care and teaching [21, 22]. However, the correlation between NIH research grant funds and Step 2 scores may also be explained by the outliers and the schools with high lev values in our dataset, which may affect regression coefficient estimates. Hence, more research should be conducted regarding this association.

Fit Diagnostics for Step 1 and 2 reveal several potential outliers (Figs. 1 and 2). University of Missouri-Columbia consistently outperforms on Step 1 and 2, despite accepting medical students with lower MCAT scores than the national average [23]. One possible explanation for the outliers may be unique features of their curriculum. Of note, curricular (i.e. early clinical exposure, minimized lecture time, and focus on clinical vignettes in a “patient-based learning” style) as well as administrative changes in 1993 to improve University of Missouri-Columbia’s medical curriculum, may have contributed to their success on the USMLE [23]. Furthermore, better exam performance may partially be explained by greater clinical exposure in the curriculum early on, where the first 2 years at the University of Missouri-Columbia are utilized for early clinical exposure and basic science education [23]. Overall, further evaluation of the curriculum at schools exceeding predictions of Step scores should be conducted to determine what is being done differently from other U.S. medical schools.

The fact the University of Missouri-Columbia is the only medical school in the United States to outperform in both Step 1 and Step 2 should draw special attention to determining what specifics of the curriculum and/or administrative organization contribute to their success. If these variables can be determined, they can be utilized at other institutions, and in turn enhance student learning. Another benefit of replicating the successes of the University of Missouri-Columbia would be that medical schools can minimize concern about board examination underperformance by students with lower than average MCAT scores, and instead place more emphasis on selecting students for admissions based on institution mission.

## Conclusions

This study uncovers several medical schools which outperform or underperform trend line expectations for USMLE, irrespective of entering student qualifications. One outlier institution, the University of Missouri-Columbia, was found to significantly outperform in both Step 1 and 2; such performance may be explained by curriculum and administrative differences. Having identified institutions that outperform expectations, the next sequence of investigations should aim to pinpoint the nuances within the “patient-based learning” curriculum that helped enhance medical education at the University of Missouri-Columbia. If these variables can be determined and disseminated, institutions globally will be able to produce physicians with greater clinical knowledge and skills, thereby improving patient care.

## Abbreviations

CK: Clinical Knowledge
GPA: Grade point average
MCAT: Medical College Admissions Test
NIH: National Institute of Health
USMLE: United States Medical Licensing Examination
USN&WR: U.S. News and World Report
